# Supplementary material for: Vitamin A supplements, routine immunization, and the subsequent risk of Plasmodium infection among children under 5 years in sub-Saharan Africa
Source: eLife. 2015 Feb 3;4:e03925. doi: 10.7554/eLife.03925 (PMC4383226; doi:10.7554/eLife.03925)
Supplement: Supplementary file 1. — Comparison of subject groups with complete and missing information on confounders. DOI: http://dx.doi.org/10.7554/eLife.03925.008 [file elife03925s001.doc]

**Supplementary File 1**. Comparison of subject groups with complete and missing information on confounders

| **Characteristic** | **Subjects with complete information on confounders**  (Included in analysis)  (n=8,413) | | **Subjects with missing**  **information on confounders**  (Excluded from analysis)  (n=3,645) | |
| --- | --- | --- | --- | --- |
|  | **Summary statistic** | **No. of missing values (%)** | **Summary statistic** | **No. of missing values (%)** |
| **Children tested for malaria**, no (%) |  |  |  |  |
| *Parasitemia* | 8,390 (99) | 23 (0.3) | 3,638 (99) | 7 (0.2) |
| Positive result among children tested for parasitemia | 9 2,312 (28) |  | 91232 (34) |  |
| *Pƒ-HRP2* | 6,121 (73) | 2292 (27) | 2,581 (71) | 1064 (29) |
| Positive results among children tested for *Pƒ-HRP2* | 92,121 (35) |  | 91,010 (39) |  |
| **Type of immunization received**, no (%) |  |  |  |  |
| *Bacille Calmette Guerin (BCG)* |  |  |  |  |
| No | 127 (1.6) | 250 (3.0) | 91 (2.6) | 158 (4.3) |
| Yes | 8,036 (98) |  | 3,396 (97) |  |
| *Diphtheria-Tetanus-Pertussis (DTP)* |  |  |  |  |
| No | 192 (2.3) | 155 (1.8) | 65 (1.8) | 103 (2.8) |
| Yes | 8,066 (96) |  | 3,477 (98) |  |
| *Measles* |  |  |  |  |
| No | 1,624 (20) | 321 (3.8) | 295 (8.6) | 197 (5.4) |
| Yes | 6,468 (80) |  | 3,153 (91) |  |
| *Poliomyelitis* |  |  |  |  |
| No | 41 (0.5) | 118 (1.4) | 24 (0.7) | 84 (2.3) |
| Yes | 8,254 (98) |  | 3,537 (99) |  |
| *Vitamin A* |  |  |  |  |
| No | 1,345 (38) | 4880 (58) | 551 (35) | 2084 (57) |
| Yes | 2,188 (62) |  | 1,010 (65) |  |
| **Confounders included in the regression model** |  |  |  |  |
| *Age in months*, median (IQR) | 22 (13-32) | 0 (0) | 44 (34-52) | 0 (0) |
| *Sex*, no (%) |  |  |  |  |
| Boys | 4,334 (52) | 0 (0) | 1,805 (50) | 0 (0) |
| Girls | 4,079 (48) |  | 1,840 (51) |  |
| *Wealth Index Score, no (%)* |  |  |  |  |
| Poorest | 1,583 (19) | 0 (0) | 765 (21) | 0 (0) |
| Poorer | 1,721 (20) |  | 808 (22) |  |
| Average | 1,719 (20) |  | 803 (22) |  |
| Wealthier | 1,768 (21) |  | 770 (21) |  |
| Wealthiest | 1,622 (19) |  | 499 (14) |  |
| *Mother’s highest educational level, no (%)* |  |  |  |  |
| None | 4,197 (50) | 0 (0) | 2,025 (56) | 0 (0) |
| Primary (incomplete) | 2,730 (32) |  | 1,172 (32) |  |
| Primary (complete) | 617 (7.3) |  | 225 (6.2) |  |
| Secondary (incomplete) | 720 (8.6) |  | 181 (5.0) |  |
| Secondary (complete) | 96 (1.1) |  | 32 (0.9) |  |
| Post-secondary | 53 (0.6) |  | 10 (0.3) |  |
| Child received antimalarial during previous week |  |  |  |  |
| *No* | 7,907 (94) | 0 (0) | 3,471 (96) | 18 (0.5) |
| *Yes* | 506 (6.0) |  | 156 (4.3) |  |
| *Child’s family owns bednet* |  |  |  |  |
| No | 1,684 (20) | 0 (0) | 714 (20) | 0 (0) |
| Yes | 6,729 (80) |  | 2,931 (80) |  |
| *Proportion of household using bednet during previous night* |  |  |  |  |
| None | 1,516 (18) | 0 (0) | 632 (18) | 42 (1.1) |
| All | 4,296 (51) |  | 1,561 (43) |  |
| Some | 917 (11) |  | 707 (20) |  |
| No bednet in household | 1,684 (20) |  | 703 (20) |  |
| *Child’s house had indoor insecticide spraying* |  |  |  |  |
| No | 5,614 (67) | 0 (0) | 2,462 (68) | 38 (1.0) |
| Yes | 711 (8.4) |  | 366 (10) |  |
| Question not part of survey questionnaire | 2,088 (25) |  | 779 (22) |  |
| *Mother’s access to antenatal care during last pregnancy* |  |  |  |  |
| No | 175 (2.1) | 0 (0) | 10 (3.8) | 3378 (93) |
| Yes | 8,238 (98) |  | 257 (96) |  |
| *Malaria transmission season* |  |  |  |  |
| Dry | 3,704 (44) | 0 (0) | 1,511 (45) | 274 (75) |
| Wet | 4,709 (56) |  | 1,860 (55) |  |
| *Type of community setting* |  |  |  |  |
| Urban | 6,203 (74) | 0 (0) | 2,858 (78) | 0 (0) |
| Rural | 2,210 (26) |  | 787 (22) |  |
