# Supplementary material for: Vitamin A supplements, routine immunization, and the subsequent risk of Plasmodium infection among children under 5 years in sub-Saharan Africa
Source: eLife. 2015 Feb 3;4:e03925. doi: 10.7554/eLife.03925 (PMC4383226; doi:10.7554/eLife.03925)
Supplement: Supplementary file 2. — Comparison of standardized bias for factors associated with vaccine/vitamin A supplement uptake, before and after inverse probability weighting (IPW). DOI: http://dx.doi.org/10.7554/eLife.03925.009 [file elife03925s002.doc]

**Supplementary File 2**. Comparison of standardized bias for factors associated with vaccine/vitamin A supplement uptake, before and after Inverse Probability Weighting (IPW)

(a)

| **Child’s characteristic** |  | **Bacille Calmette Guerin (BCG)** | | | | **Diphtheria-Tetanus-Pertussis (DTP)** | | | |
| --- | --- | --- | --- | --- | --- | --- | --- | --- | --- |
|  |  | **Not Vaccinated** | **Vaccinated** | **Standardized Bias** | | **Not Vaccinated** | **Vaccinated** | **Standardized Bias** | |
|  |  |  |  | **Before IPW** | **After IPW** |  |  | **Before IPW** | **After IPW** |
| *Age*, mean (SD) |  | 15.52 (14.2) | 19.98 (14.0) | **-0.94** | +0.15 | 5.76 (9.2) | 21.21 (13.7) | **-0.78** | **+0.51** |
| *Mother’s Age*, mean (SD) |  | 28.77 (7.4) | 28.81 (7.2) | **-1.23** | +0.02 | 27.34 (7.0) | 28.92 (7.2) | -0.06 | +0.11 |
| *Gender*, no(%) | Girls | 182 (48) | 6,863 (49) | ±0.03 | ±0.01 | 603 (48) | 6,580 (49) | ±0.03 | ±0.03 |
|  | Boys | 198 (52) | 7,073 (51) |  |  | 650 (52) | 6,726 (50) |  |  |
| *Low Birthweight*, no(%) | No | 151 (40) | 8,920 (64) | **±0.49** | ±0.03 | 741 (59) | 8,457 (64) | ±0.09 | ±0.10 |
|  | Yes | 229 (60) | 5,016 (36) |  |  | 512 (41) | 4,849 (36) |  |  |
| *Presence of radio and/or TV in household*, no(%) | No | 149 (39) | 4,325 (31) | ±0.17 | ±0.01 | 405 (32) | 4,088 (31) | ±0.03 | ±0.06 |
|  | Yes | 237 (62) | 9,794 (70) |  |  | 848 (68) | 9,218 (69) |  |  |
| *Type of community setting*, no(%) | Urban | 307 (81) | 9,807 (70) | ±0.24 | ±0.12 | 922 (74) | 9,341 (70) | ±0.08 | ±0.05 |
|  | Rural | 73 (19) | 4,129 (30) |  |  | 331 (26) | 3,965 (30) |  |  |
| *Breastfeeding status*, no(%) | No | 104 (27) | 5,096 (37) | ±0.20 | ±0.09 | 100 (8.0) | 5,201(39) | **±0.73** | **±0.90** |
|  | Yes | 276 (73) | 8,840 (63) |  |  | 1,153 (92) | 8,105 (61) |  |  |
| *Wealth Index Score*, no(%) | Poorer | 111 (29) | 2,442 (18) | **+0.28** | +0.00 | 257 (20) | 2,326 (18) | +0.08 | +0.10 |
|  | Poor | 100 (26) | 2,875 (21) | +0.13 | +0.00 | 284 (23) | 2,734 (20) | +0.05 | +0.06 |
|  | Average | 76 (20) | 2,970 (21) | -0.03 | -0.07 | 288 (23) | 2,812 (21) | +0.04 | +0.02 |
|  | Rich | 52 (14) | 2,982 (21) | -0.20 | -0.05 | 224 (18) | 2,869 (22) | -0.09 | -0.06 |
|  | Richer | 41 (11) | 2,667 (19) | -0.23 | +0.11 | 200 (16) | 2,565 (19) | -0.09 | -0.12 |
| *Mother’s highest education level*, no(%) | None | 182 (48) | 6,121 (44) | +0.08 | -0.04 | 623 (50) | 5,735 (43) | +0.13 | +0.10 |
|  | Primary (incomplete) | 154 (40) | 4,460 (32) | +0.18 | +0.01 | 391 (31) | 4,331 (32) | -0.03 | -0.04 |
|  | Primary (completed) | 17 (4.5) | 1,299 (9.3) | -0.19 | +0.02 | 85 (6.8) | 1,263 (9.5) | -0.10 | -0.07 |
|  | Secondary (incomplete) | 24 (6.3) | 1,703 (12) | -0.20 | +0.02 | 136 (11) | 1,624 (12) | -0.04 | -0.03 |
|  | Secondary (completed) | 0 (0) | 186 (1.3) | -0.16 | -0.16 | 11 (0.8) | 188 (1.4) | -0.05 | -0.01 |
|  | Post-secondary | 3 (0.8) | 167 (1.2) | -0.04 | 0.12 | 7 (0.6) | 165 (1.2) | -0.07 | -0.02 |
| *Antenatal care during last pregnancy*, no(%) | No | 47 (12) | 351 (2.5) | **±0.38** | ±0.01 | 63 (5.0) | 343 (2.6) | ±0.13 | ±0.11 |
|  | Yes | 333 (88) | 13,585 (98) |  |  | 1,190 (95) | 12,963 (97) |  |  |
| *Mother’s tetanus status*, no(%) | No | 98 (26) | 1,923 (14) | **±0.30** | ±0.03 | 218 (17) | 1,846 (14) | ±0.10 | ±0.06 |
|  | Yes | 282 (74) | 12,013 (86) |  |  | 1,035 (83) | 11,460 (86) |  |  |

(b)

| **Child’s characteristic** |  | **Measles** | | | | **Polio** | | | |
| --- | --- | --- | --- | --- | --- | --- | --- | --- | --- |
|  |  | **Not Vaccinated** | **Vaccinated** | **Standardized Bias** | | **Not Vaccinated** | **Vaccinated** | **Standardized Bias** | |
|  |  |  |  | **Before IPW** | **After IPW** |  |  | **Before IPW** | **After IPW** |
| *Age*, mean (SD) |  | 8.32 (8.7) | 26.05 (12.4) | **-1.26** | **+0.56** | 8.07 (11.8) | 20.07 (14.0) | **-0.85** | **+0.65** |
| *Mother’s Age*, mean (SD) |  | 27.47 (7.0) | 29.48 (7.2) | **-0.28** | +0.14 | 27.70 (7.3) | 28.80 (7.2) | -0.15 | +0.02 |
| *Gender*, no(%) | Girls | 2,513 (50) | 4,521 (49) | ±0.03 | **±0.34** | 115 (48) | 7,097 (49) | ±0.02 | **±0.36** |
|  | Boys | 2,497 (50) | 4,756 (51) |  |  | 123 (52) | 7,282 (51) |  |  |
| *Low Birthweight*, no(%) | No | 3,005 (60) | 6,025 (65) | ±0.10 | **±0.28** | 129 (54) | 9,101 (63) | ±0.18 | ±0.20 |
|  | Yes | 2,005 (40) | 3,252 (35) |  |  | 109 (46) | 5,278 (37) |  |  |
| *Presence of radio and/or TV in household*, no(%) | No | 1,643 (33) | 2,781 (30) | ±0.06 | ±0.09 | 79 (33) | 4,431 (31) | ±0.05 | **±0.71** |
|  | Yes | 3,367 (67) | 6,496 (70) |  |  | 159 (67) | 9,948 (69) |  |  |
| *Type of community setting*, no(%) | Urban | 3,612 (72) | 6,463 (70) | ±0.05 | ±0.19 | 169 (71) | 10,126 (70) | ±0.01 | **±1.17** |
|  | Rural | 1,398 (30) | 2,814 (30) |  |  | 69 (29) | 4,253 (30) |  |  |
| *Breastfeeding status*, no(%) | No | 476 (9.5) | 4,691 (51) | **±0.90** | **±0.89** | 32 (13) | 5,292 (37) | **±0.54** | **±1.41** |
|  | Yes | 4,534 (90) | 4,586 (49) |  |  | 206 (86) | 9,087 (63) |  |  |
| *Wealth Index Score*, no(%) | Poorer | 1,010 (20) | 1,520 (16) | +0.10 | **-0.72** | 49 (21) | 2,544 (18) | +0.07 | **-0.82** |
|  | Poor | 1,117 (22) | 1,849 (20) | +0.06 | -0.27 | 53 (22) | 2,973 (21) | +0.04 | **-0.38** |
|  | Average | 1,089 (22) | 1,933 (21) | +0.02 | +0.21 | 59 (25) | 3,047 (21) | +0.09 | **-0.62** |
|  | Rich | 988 (20) | 2,051 (22) | -0.06 | +0.01 | 36 (15) | 3,075 (21) | -0.16 | **+0.80** |
|  | Richer | 806 (16) | 1,924 (21) | -0.12 | **+0.27** | 41 (17) | 2,740 (19) | -0.05 | +0.08 |
| *Mother’s highest education level*, no(%) | None | 2,348 (47) | 3,891 (42) | +0.10 | +0.14 | 108 (45) | 6,208 (43) | +0.04 | **-0.71** |
|  | Primary (incomplete) | 1,570 (31) | 3,076 (33) | -0.04 | -0.04 | 85 (36) | 4,653 (32) | +0.07 | **-1.06** |
|  | Primary (completed) | 402 (8.0) | 919 (9.9) | -0.07 | +0.10 | 12 (5.0) | 1,337 (9.3) | -0.16 | **-0.42** |
|  | Secondary (incomplete) | 572 (12) | 1142 (12) | -0.03 | -0.27 | 31 (13) | 1,738 (12) | +0.03 | +0.22 |
|  | Secondary (completed) | 63 (1.3) | 132 (1.4) | -0.01 | -0.05 | 1 (4.2) | 199 (1.4) | -0.10 | **+1.11** |
|  | Post-secondary | 55 (1.1) | 117 (1.3) | -0.02 | -0.05 | 1 (4.2) | 172 (1.2) | -0.09 | -0.03 |
| *Antenatal care during last pregnancy*, no(%) | No | 201 (4.0) | 198 (2.1) | ±0.11 | ±0.12 | 24 (10) | 384 (2.7) | **±0.30** | ±0.20 |
|  | Yes | 4,809 (96) | 9,079 (98) |  |  | 214 (90) | 13,995 (97) |  |  |
| *Mother’s tetanus status*, no(%) | No | 777 (15) | 1,255 (14) | ±0.06 | ±0.03 | 58 (24) | 2,015 (14) | **±0.26** | ±0.22 |
|  | Yes | 4,233 (85) | 8,022 (86) |  |  | 180 (76) | 12,364 (86) |  |  |

(c)

| **Child’s characteristic** |  | **Vitamin A** | | | |
| --- | --- | --- | --- | --- | --- |
|  |  | **Not Vaccinated** | **Vaccinated** | **Standardized Bias** | |
|  |  |  |  | **Before IPW** | **After IPW** |
| *Age*, mean (SD) |  | 12.28 (12.6) | 24.20 (13.0) | **-0.84** | +0.20 |
| *Mother’s Age*, mean (SD) |  | 27.83 (7.1) | 28.81 (7.2) | -0.14 | +0.07 |
| *Gender*, no(%) | Girls | 1,950 (49) | 2,380 (50) | ±0.01 | ±0.01 |
|  | Boys | 2,000 (51) | 2,375 (50) |  |  |
| *Low Birthweight*, no(%) | No | 2,319 (59) | 3,067 (64) | ±0.12 | ±0.04 |
|  | Yes | 1,631 (41) | 1,688 (35) |  |  |
| *Presence of radio and/or TV in household*, no(%) | No | 1,307 (33) | 1,520 (32) | ±0.02 | ±0.01 |
|  | Yes | 2,643 (67) | 3,235 (68) |  |  |
| *Type of community setting*, no(%) | Urban | 2,832 (72) | 2,985 (63) | ±0.19 | ±0.06 |
|  | Rural | 1,118 (28) | 1,770 (37) |  |  |
| *Breastfeeding status*, no(%) | No | 790 (20) | 2,507 (53) | **±0.68** | ±0.18 |
|  | Yes | 3,160 (80) | 2,248 (47) |  |  |
| *Wealth Index Score*, no(%) | Poorer | 803 (20) | 685 (14) | +0.16 | -0.02 |
|  | Poor | 914 (23) | 891 (19) | +0.11 | -0.05 |
|  | Average | 824 (21) | 953 (20) | +0.02 | -0.05 |
|  | Rich | 742 (19) | 1,094 (23) | -0.10 | +0.02 |
|  | Richer | 667 (17) | 1,132 (24) | -0.17 | +0.09 |
| *Mother’s highest education level*, no(%) | None | 1,873 (47) | 1,335 (28) | **+0.40** | -0.02 |
|  | Primary (incomplete) | 1,153 (29) | 1,817 (38) | -0.19 | -0.01 |
|  | Primary (completed) | 334 (8.4) | 600 (13) | -0.14 | +0.03 |
|  | Secondary (incomplete) | 491 (12) | 808 (17) | -0.13 | +0.04 |
|  | Secondary (completed) | 44 (1.1) | 103 (2.2) | -0.08 | -0.06 |
|  | Post-secondary | 55 (1.4) | 92 (1.9) | -0.04 | -0.03 |
| *Antenatal care during last pregnancy*, no(%) | No | 176 (4.5) | 128 (2.7) | ±0.10 | ±0.02 |
|  | Yes | 3,774 (95) | 4,627 (97) |  |  |
| *Mother’s tetanus status*, no(%) | No | 591 (15) | 721 (15) | ±0.01 | ±0.03 |
|  | Yes | 3,359 (85) | 4,034 (85) |  |  |
