# Supplementary material for: Vitamin A supplements, routine immunization, and the subsequent risk of Plasmodium infection among children under 5 years in sub-Saharan Africa
Source: eLife. 2015 Feb 3;4:e03925. doi: 10.7554/eLife.03925 (PMC4383226; doi:10.7554/eLife.03925)
Supplement: Supplementary file 3. — Overview of immunization schedules for each country. DOI: http://dx.doi.org/10.7554/eLife.03925.010 [file elife03925s003.doc]

**Supplementary File 3. Overview of Immunization Schedules for each country^1^**

| **Type of Vaccination/**  **Supplementation** | **Country** | | | |
| --- | --- | --- | --- | --- |
|  | **Burkina Faso** | **Mozambique** | **Rwanda** | **Senegal** |
| *BCG* | Birth | Birth | Birth | Birth |
| *DTP* | 8,12,16 weeks*  (DTP) | 6,10,14 weeks (DTPHep) | 6,10,14 weeks  (DTPHibHep) | 6,10,14 weeks  (DTPHibHep) |
| *Measles* | 9 months | 9 months | 9 months | 9 months |
| *Polio* | Birth,8,12,16 weeks* | 6,10,14 weeks | Birth, 6,10,14 weeks | Birth, 6,10,14 weeks |
| *Vitamin A* | Every 6 months from ages 6-59 months  *Main Distribution Mechanism*:  Integrated Child Health campaign | Every 6 months from ages 6-59 months  *Main Distribution Mechanism*:  Integrated Child Health campaign | Every 6 months from ages 6-59 months  *Main Distribution Mechanism*:  Integrated Child Health campaign | Every 6 months from ages 6-59 months  *Main Distribution Mechanism*:  Integrated Child Health campaign |

*Guidelines report immunization schedule in months (2,3,4 months). Converted into weeks for this table.

^1^Adapted from: Unicef and WHO. Immunization Summary: The 2007 Edition. (published February 2007), Vaccination Information Management System: [www.vimsdata.org](http://www.vimsdata.org/) (Last accessed September 19, 2014) and personal communication with E. Alden (UNICEF) on December 8, 2014.
